# Editorial: Insights in integrative neuroscience: 2021

**DOI:** 10.3389/fnint.2022.1084091

**Published:** 2022-11-28

**Authors:** Elizabeth B. Torres

**Affiliations:** Psychology Department, Rutgers Center for Cognitive Science and Rutgers Center for Computational Biomedicine Imaging and Modelling, Computer Science Department, Rutgers, The State University of New Jersey, Piscataway, NJ, United States

**Keywords:** human birth, light, posterior parietal cortex, multisensory integration, sevoflurane, RNA sequencing, cannabis, primate welfare

In the last decade, Neuroscience has made large strides across disciplines. We have witnessed a surge in new instrumentation and innovative analytical techniques, paired with collaborative interdisciplinary work. These advances in scientific practices have been fueled by open access and integration of knowledge across different levels of inquiry. Along these lines, Integrative Neuroscience is beginning to play a key role in providing a bird's view of new emerging ideas, new insights across disciplines, current challenges, and future perspectives across sensory-motor processing, perceptual sciences, and Neurophysiology.

This collection of papers highlights recent developments, major accomplishments, and transformative views poised to move the field forward. They provide an overview on the state-of-the-art discoveries and future challenges across different areas of basic science and applied, translational research, thus updating several traditional stances and providing guidance for future endeavors to researchers across multiple subfields of Neuroscience.

Polese et al. uncover important issues in developmental neuroscience examining the dynamics involved in human birth. Their hypothesis and theory manuscript “*The newborn's reaction to light as the determinant of the brain's activation at human birth”* posits that light is a fundamental form of sensory input characterized by novelty, efficacy, ubiquity, and immediacy and a good candidate for initiating a sudden brain shift from the prenatal to neonatal patterns of functions. The authors propose in their review that this theorized important and novel function of light for switching brain states at birth, could also trigger a broad range of diversified research across different domains, spanning from neurophysiology to neurology and psychiatry.

Vaccari et al. write an impactful minireview entitled “*New insights on single-neuron selectivity in the era of population-level approaches.”* They highlight the increasing need for considering multiplexing roles for single neurons participating as fundamental units of neuronal ensembles that are today simultaneously accessible, owing to recent technological advances. They review and contrast traditional concepts of single cell functional selectivity specialized on the computation of a single stimulus feature, with the more recent view of mixed selectivity considering multiplexing in single neurons encoding multiple features and recruited on demand. The authors review evidence consistent with these concepts in the posterior parietal, the motor, and the prefrontal cortex, thus offering support for the more recently emergent views and arguing for a more efficient code than that proposed by the traditional single cell receptive field view.

Cheng et al. report original research results in their manuscript entitled “*Identification of prefontal cortex and amygdala expressed genes associated with sevoflurane anesthesia on non-human primate.”* The authors use transcriptional studies and bioinformatics analyses to forward our understanding of the primate prefrontal cortex (PFC) and amygdala following the use of sevoflurane anesthesia in early neurodevelopmental stages. The study is very important as this anesthesia is broadly used in human infancy, without clear understanding of potential neurotoxic effects on vital encephalic regions such as the amygdala. Using a non-human primate (macaque) model, they report that in comparison with the amygdala's changing pattern following sevoflurane exposure, functional annotations of the PFC were more enriched in glial cell-related biological functions than in neuron and synapsis development. They conclude that these transcriptome changes in the specimens that they analyzed support the relevant role of the amygdala in the biological processes influenced by sevoflurane and may advance our understanding of neuronal injury caused by sevoflurane.

Banks et al. report original research results in their paper entitled “*Cannabis use is associated with sexually dimorphic changes in executive control of visuospatial decision-making.”* They studied key aspects of cognitive control in men and women who use cannabis frequently within the context of a two-choice decision-making paradigm that varied spatial location and visual features (color) of the stimuli. In their study, they assess the extent to which people who report frequent use of cannabis would shift their choice in the face of an incorrect outcome -a strategy coined lose-shift. They found that the spatial position of choice targets drives the lose-shift effect, with marked differences between men and women user of cannabis. More precisely, viewed from the perspective of a reinforcement learning paradigm, the executive function to inhibit lose-shift responding to gain reward is different among male and female habitual cannabis users. In women, cannabis use suppresses response flexibility, paired with an increased tendency to lose-shift. This in turn reduces performance in a choice task in which random responding is the optimal strategy. On the other hand, increased cannabis use in men appears to be congruent with reduced reliance on spatial cues during decision-making and had no impact on accuracy. Their work provides compelling evidence that spatial-motor processing is an important component of economic decision-making, and that its governance by executive systems is different in men and women who use cannabis frequently.

In a hypothesis theory paper, Iriki and Tramacere propose novel strategies for primate experimentation in Natural Labs that enhance ethical value and have higher utility for cognitive neuroscience and neuropsychiatric research. Their paper entitled “*Natural Laboratory Complex for novel primate neuroscience”* is an example of innovative ideas congruent with the new challenges of twenty-first century integrative neuroscience. In this work, they propose a combination of indoor and outdoor facilities whereby the animals can move freely and interact socially in ways amenable to study their natural behaviors. Their proposition is that such natural labs would promote ecological validity to future studies and significantly improve primate welfare. Furthermore, they explain how recent advances enabling remote infrastructure could facilitate the implementation of monitoring methods that would forward our understanding of more naturalistic behaviors. The ethical and economic benefits of their proposition are indeed undisputable.

A paper by Bauer et al. entitled “*Validation of functional connectivity of engineered neuromuscular junction with recombinant monosynaptic pseudotyped* Δ*G-rabies virus tracing”* is an excellent example of new methods to advance pre-clinical models of neurodegenerative diseases, whereby *in vitro* neural engineering approaches enable the selective study of relevant neuronal classes, networks, and functional units to probe new hypotheses about neurodegeneration. They provide an example with amyotrophic lateral sclerosis (ALS) whereby *in vitro* models of the neuromuscular junction (NMJ) are amenable to test whether motor neuron degeneration in ALS starts at the nerve terminal or at the NMJ and retrogradely progresses to the motor neuron cell body. This work is a perfect example of interdisciplinary collaboration integrating multiple bodies of knowledge whereby the total outcome is much more than the sum of its components.

Last, but equally important is an ongoing debate in the field of clinical interventions where in a general commentary article Schoen et al. respond to an article by Camarata et al. (2020) about “*Evaluating Sensory Integration/Sensory Processing treatment: Issues and Analyses*.”

In their commentary Schoen et al. argue that in their paper, Camarata et al. (2020) inaccurately characterized the intervention components, employed language not used in the field, and proposed an inappropriate framework for systematic testing. The authors advocate for consistency in the treatments and outcome measures to strengthen the existing evidence base. This continuing conversation is important to move the clinical field forward at a time when neurodevelopmental disorders are on the rise.

This Research Topic brought a variety of forward-thinking work outlining current challenges and suggesting transformative strategies that will advance basic research in Integrative Neuroscience from molecules to complex social behaviors along with translational applications to the many pressing medical issues of our times.

## Author contributions

The author confirms being the sole contributor of this work and has approved it for publication.

## Funding

Funding was provided to ET by the New Jersey Governor's Council for Autism Research Grant # CAUT18ACE014.

## Conflict of interest

The author declares that the research was conducted in the absence of any commercial or financial relationships that could be construed as a potential conflict of interest.

## Publisher's note

All claims expressed in this article are solely those of the authors and do not necessarily represent those of their affiliated organizations, or those of the publisher, the editors and the reviewers. Any product that may be evaluated in this article, or claim that may be made by its manufacturer, is not guaranteed or endorsed by the publisher.
